# Weather parameters as a predictive tool potentially allowing for better monitoring of dairy cattle against gastrointestinal parasites hazard

**DOI:** 10.1038/s41598-023-32890-0

**Published:** 2023-04-12

**Authors:** Katarzyna Płoneczka-Janeczko, Wiwiana Szalińska, Irena Otop, Jolanta Piekarska, Krzysztof Rypuła

**Affiliations:** 1 Department of Epizootiology with Clinic for Birds and Exotic Animals, Faculty of Veterinary Medicine, Wroclaw University of Environmental and Life Sciences, Grunwaldzki Square 45, 50-366 Wrocław, Poland; 2 Research and Development Centre, Institute of Meteorology and Water Management – National Research Institute, Podleśna 61, 01-673 Warsaw, Poland; 3 Division of Parasitology, Department of Internal Medicine and Clinic of Diseases of Horses, Dogs and Cats, Faculty of Veterinary Medicine, Wrocław University of Environmental and Life Sciences, Grunwaldzki Square 47, 50-366 Wrocław, Poland

**Keywords:** Climate sciences, Environmental sciences, Diseases

## Abstract

In animal production, yield is critically related to animal health status. To ensure high productivity, innovative control strategies for herd and parasites monitoring are required. Gastrointestinal parasites have a strong influence on changing feed intake or nutrient use, limiting animal productivity. Serological control has been proposed, given that parasite development is largely dependent on environmental temperature and humidity. However, breeders and field veterinarians lack readily accessible climate characteristics that provide information to determine whether and when herds require laboratory examination. To help reduce the testing costs incurred by farmers, we investigated whether selected meteorological data could serve as conclusive predictors to increase the precision of herd selection for serological monitoring. Our results indicate that the selection of herds by farmers for testing can be guided by regular checking of meteorological data, especially various temperature and humidity indicators. In general, ranges of 24–28 °C, as well as − 0.5 to 7.5 °C for the monthly maximum and minimum temperature, respectively, and relative humidity (68–79%) and vapour pressure (10–15 hPa) correspond to a high antiparasitic response of the herd, expressed as the optical density ratio. It is recommended to introduce coproscopic and/or serological tests if the observed weather pattern (covering the prepatent period of parasite development) ranges within the estimated values.

## Introduction

Although climatic dependencies and their relationships with human diseases have been studied for years, the topic remains open in veterinary medicine, especially with respect to livestock and their management. These associations are multifaceted. Animal production contributes to atmospheric warming through green-house gas (GHG) emission. On the other hand, most livestock diseases, heat stress, and the availability, quality, and composition (biodiversity) of grazing areas are climate dependent^1^. Due to the high sensitivity of plant production to fluctuations in climatic conditions, animal feeding and productivity remain a popular research topic.

According to the World Meteorological Organisation, the past 7 years were the warmest on record^2^. Climate changes widely described on a global scale include the alteration of the composition of the atmosphere, that is, the accumulation of greenhouse gases (GHG), intense rainfall and flooding, corresponding to dairy production^3–5^. The climate crisis also affects the transmission of disease agents, causing a shift in the prevalence of parasitic, water-borne, and vector-borne diseases^6,7^. The possibility that climate change alters disease dynamics and promotes or exacerbates outbreaks in humans and animals has inspired several reviews on these potential effects^7,8^. Poland is located in a transitional zone between the maritime western and continental eastern European climates, resulting in the high variability of the weather and significant seasonal differences from year to year due to frequent air mass collision. The variability of the thermal conditions in Poland is consistent with the trends observed in recent decades in Europe and the rest of the world, and according to climate projections for the Representative Concentration Pathway (RCP) 4.5 scenario, the average annual temperature in Poland will probably increase by 1.1 °C before 2050 and by 2 °C during the period 2071 to 2100^9,10^. All these climate changes affect the alteration of host-parasite dynamics also in veterinary medicine^11^.

The Organisation for Economic Cooperation and Development and the Food and Agriculture Organisation predict that global milk production will increase from 2020 to 2029 by an estimated 1.6% to 997 million tons, and the European Union is one of the three major global dairy exporters^2^. Given the effects of dairy production on the environment and climate, including through GHG emission, it would be appropriate to reach this estimated level through the improvement and maintenance of high dairy cattle productivity, rather than through a permanent increase in the global cattle population. However, the requirement to provide a high-quality health status of dairy cattle generates additional costs related to laboratory tests for infectious and parasitic diseases. Cattle parasites constitute a major problem, reducing production and causing financial losses due to their control and the treatment and mortality of affected animals. The parasite status of the site strongly corresponds to the environmental conditions. The Intergovernmental Panel on Climate Change reported that rising global temperatures, the increase in the number of days with high daytime and nighttime temperatures, and the decrease in the number of days with temperatures < 0 °C, together with changes in rainfall patterns or prolonged drought, strongly affect the of parasitic diseases^3^. The effects of parasites often persist throughout the lactation period, although most of the parasite burdens from cattle are subclinical, with no clear or visible sign^12^. Rashid et al.^13^ estimated that the average decrease in milk production attributable to general overall parasitic infections of cattle was 1.16 L/cow/day, representing $50.67/animal/year. The most economically important parasites include protozoans and helminths, with nematodes playing a significant role. In 2020, Charlier et al.^14^ estimated that the annual cost related to helminths in the dairy cattle sector was 941 million euros.

Most parasite control measures are based on the use of anthelmintic drugs or the improvement of pasture management. Another option is the rapid identification of worms in herds or individuals by laboratory testing; coproscopic analysis, and serological identification^15^. As the impacts of climate parameters and changes therein are best exemplified by the occurrence of vector-borne or parasitic diseases in humans and animals, compound climate analysis may be a control strategy that reduces the costs of the monitoring and laboratory testing^16–20^. An understanding of the relationship between the parasite life cycle in the environment and risk factors for parasite viability outside the host species (especially temperature and humidity) is crucial to address invasion.

Most data on requirements for the free-living, development stages of parasites originate from traditional experimental laboratory research, in which fecal cultures have been processed in different ranges of moisture and temperature under laboratory conditions^21^. Modelling the potential effect of environmental conditions on the dynamics of the larval ostertagia population is also available^22^. To reduce the cost and amount of laboratory testing, innovative multidisciplinary scientific research has been undertaken. For example, the normalised difference vegetation index, obtained by satellite remote sensing (RS), was used to develop predictive and risk maps for human fasciolosis^23^. Furthermore, various geographic information systems (GIS) and climatic data, climate forecast indices, and remote sensing (RS) data were incorporated for multidisciplinary analysis of human and animal fasciolosis epidemiology, with results from a regional model extrapolated to other endemic areas^23,24^. This experience with combined GIS, RS, and spatial analysis in human health research could be extended to veterinary medicine. The application of such methods could increase the efficiency and sustainability of domestic animal parasite control programmes^25–27^.

Although the relationship between environmental factors and parasite transmission is complex, climate parameters might serve as predictors of the need for laboratory-based herd monitoring. Ostertagiosis (*Ostertagia ostertagi*), a globally distributed parasitosis, can serve as a climate-dependent disease model^28^. The available data on ostertagiosis in Poland are limited and fragmentary. Research carried out by Rypuła et al.^29^ from the two different economic regions of Poland (corresponding approximately with eastern and western Poland ) indicated that the mean ODR and SD was 0.421/0.157 and 0.483/0.236, respectively. Kowalczyk et al.^30^ estimated the herd level seroprevalence of *Ostertagia ostertagii* infection in central and north-eastern provinces of Poland. The result of the mean ODRs obtained in this study varies from 0.010 to 1.080 in the north-eastern provinces and was significantly higher than ODR in the central provinces, where ranges from 0.010 to 1.040. Our previous study from the Lower Silesia region of Poland revealed the mean ODR value of 0.53 (the ODR was between – 0.088 and 1.024)^31^.

Serological testing is a recommended control strategy for monitoring of the invasion in dairy herds. The aim of this study was to determine whether selected and readily available meteorological data serve as predictors of conditions that increase the risk of parasitosis invasion, and to increase the precision of cattle herd selection for serological monitoring.

## Results

**Figure 1:**
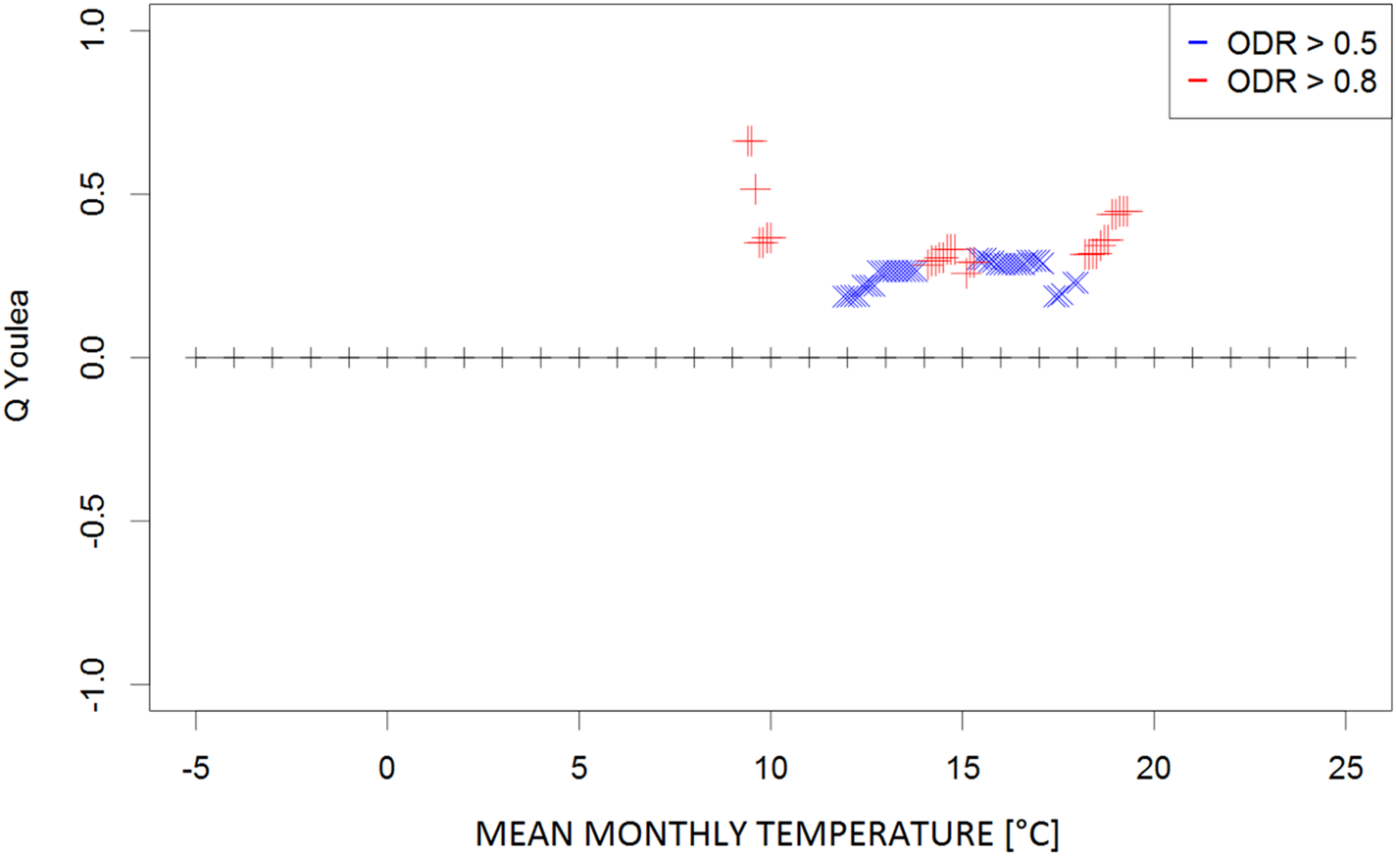
Plot of Q coefficients for different threshold levels of mean monthly temperature (Ta) for the investigated ODR limits (≥ 0.5 and ≥ 0.8).

**Figure 2:**
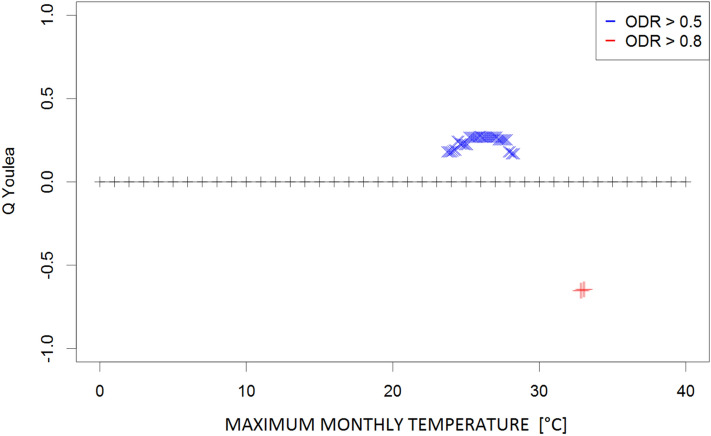
Plot of Q coefficients for different threshold levels of monthly absolute maximum temperature (Tx_ab) for the investigated ODR limits (≥ 0.5 and ≥ 0.8).

**Figure 3:**
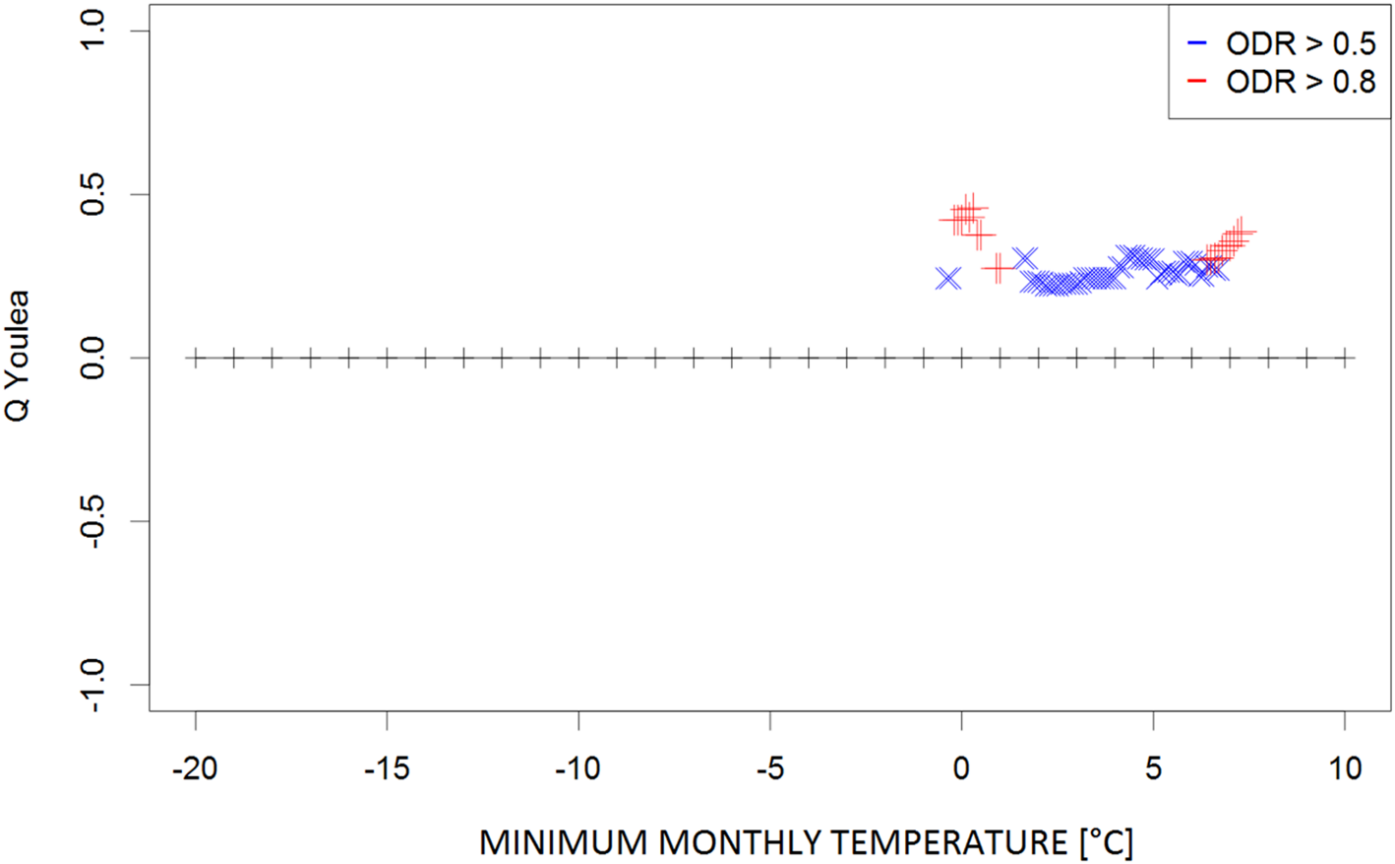
Plot of Q coefficients for different threshold levels of the monthly absolute minimum temperature (Tn_ab) for the investigated ODR limits (≥ 0.5 and ≥ 0.8).

**Figure 4:**
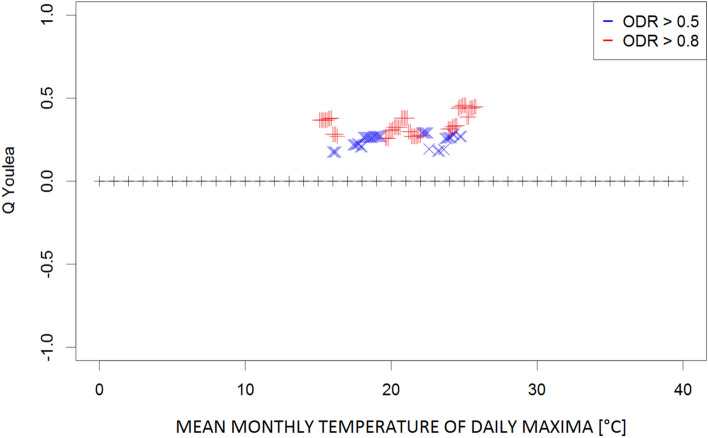
Plot of Q coefficients for different threshold levels of the mean monthly temperature of daily maxima (Tx) for the investigated ODR limits (≥ 0.5 and ≥ 0.8).

**Figure 5:**
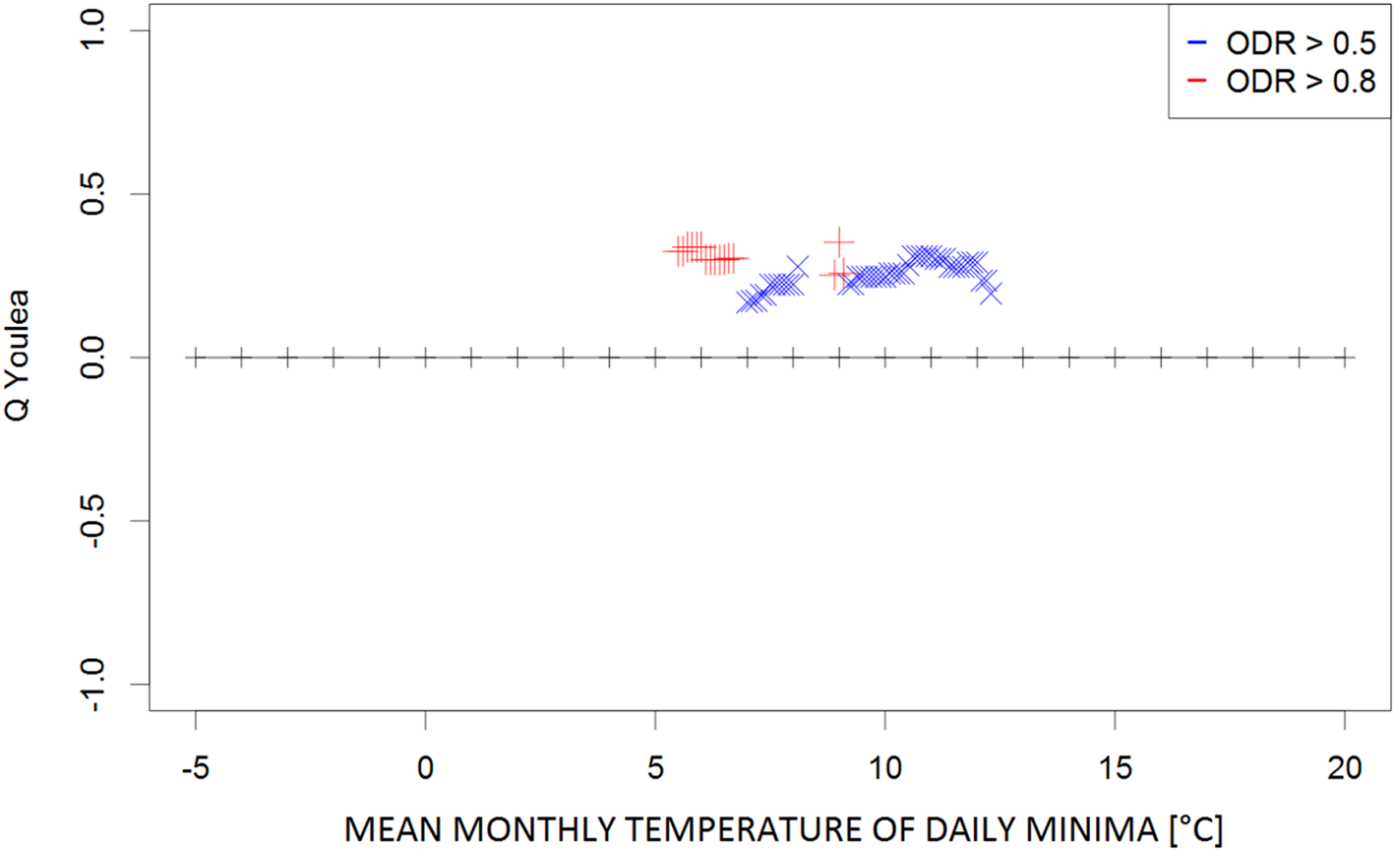
Plot of Q coefficients for different threshold levels of monthly temperature of daily minima (Tn) for the investigated ODR limits (≥ 0.5 and ≥ 0.8).

**Figure 6:**
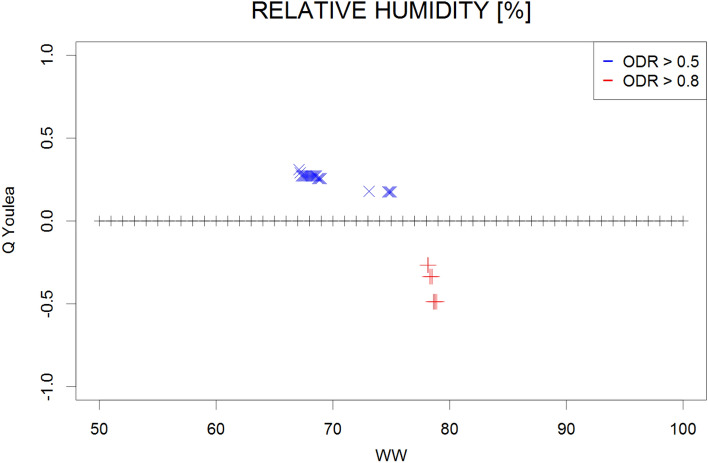
Plot of Q coefficients for different threshold levels of relative humidity (RH) for the investigated ODR limits (≥ 0.5 and ≥ 0.8).

**Figure 7:**
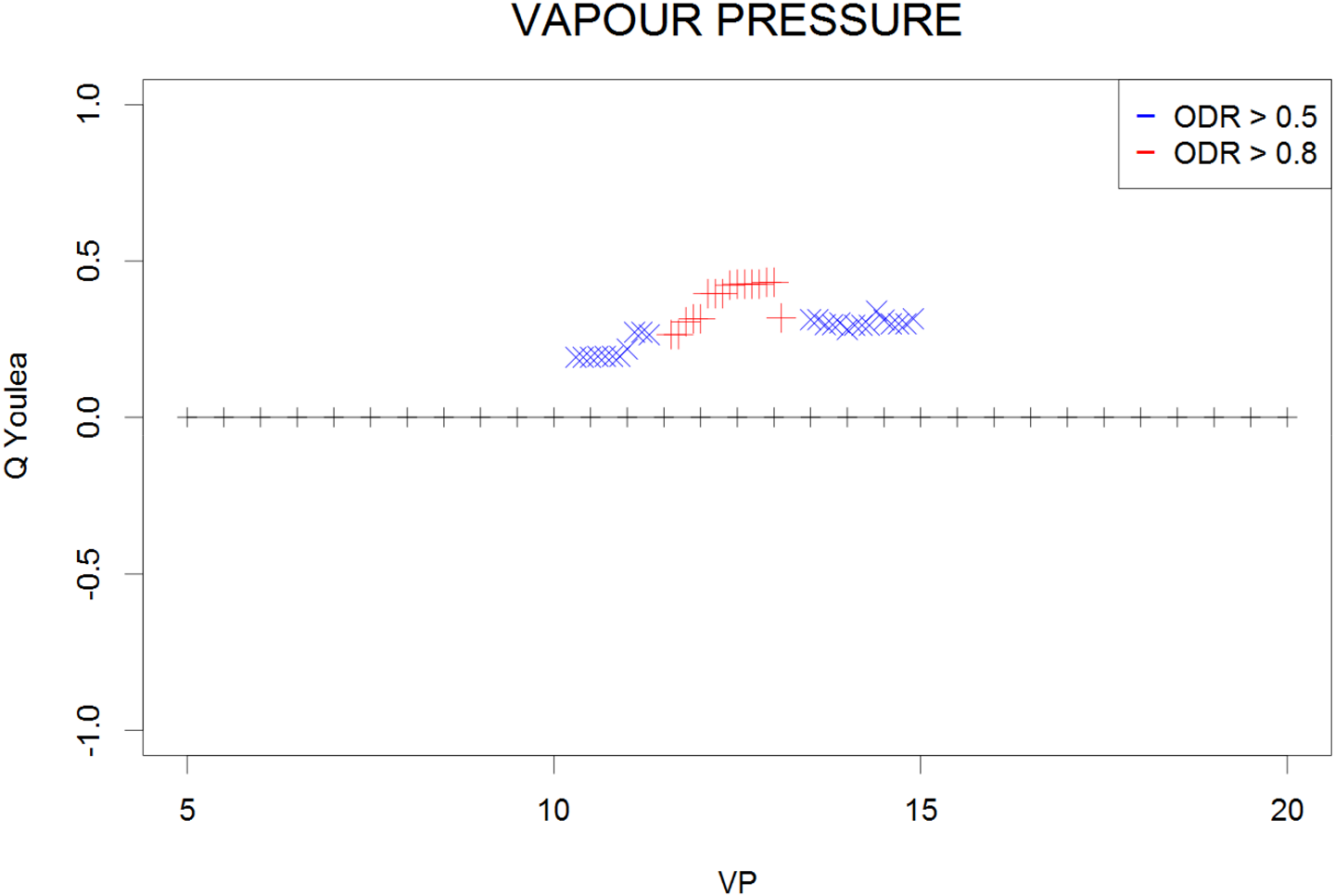
Plot of Q coefficients for different threshold levels of vapour pressure (VP) for the investigated ODR limits (≥ 0.5 and ≥ 0.8).

The Q contingency coefficients obtained for the mean, maximum, and minimum monthly temperatures; mean monthly temperatures of the daily maxima and minima; and the mean monthly relative humidity and vapour pressure are presented in Figs. 1, 2, 3, 4, 5, 6 and 7, respectively. Values represent the dependency between the high (≥ 0.5) and very high (≥ 0.8) the optical density ratio (ODR) values and the parameter threshold values.

Due to the complexity of the phenomena, the dependence strength was moderate (≤ 0.4) in most cases, indicating that the prevailing meteorological conditions are not the only factors determining the transmission of parasites in the environment. However, the consistency of the patterns (i.e., continuity of Q values along the threshold gradients) supports the identification of ranges for the respective climatological conditions that potentially contribute to such transmission. These ranges were 9–20 °C for the monthly mean temperature (Fig. 1) and 24–28 °C for the monthly maximum temperature (corresponding to high ODR values; Fig. 2); maximum temperatures > 33 °C showed a strong negative correspondence with very high ODR values. Monthly minimum temperatures between − 0.5 °C and 7.5 °C and mean daily maxima of 15–26 °C corresponded to high and very high ODR values (Figs. 3, 4). For the mean monthly temperatures of the daily minima, the best correspondence was in the range of 5.5–12.5 °C (Fig. 5). The relative humidity and vapour pressure ranges that affected the ODR values were 68–79% and 10–15 hPa, respectively (Figs. 6, 7).

## Discussion and conclusions

The climate of Poland is characterised by a high variability of the weather conditions. The parallel pattern of the main landform types allows free zonal circulation and clash of oceanic and continental air masses. Furthermore, mountainous and upland areas in the south of Poland contribute to local modifications of weather conditions. In the north, the influence of the Baltic Sea affects the pattern of meteorological elements.

During the period 1991–2020, the average annual air temperature in non-mountain areas varied from approximately 7 °C in northeastern to > 9.5 °C in western Poland^32^. The average annual precipitation is approximately 600 mm in the lowlands and exceeds 1300–1500 mm in mountainous areas of the country. About 40% of annual precipitation is recorded from June to August, including the cattle pasture season. Climate change in Poland manifests itself mainly as a significant increase in air temperature. Temperature extremes (especially in summer) have become more intense and affect a larger portion of Poland^33^. The maximum summer temperature increased by 0.4 °C per 10 years during 1951–2015, and positive trends have been observed in other indices that describe extreme temperature events (i.e., more frequent, long-lasting, and intense heat waves)^33–35^.

**Figure 8:**
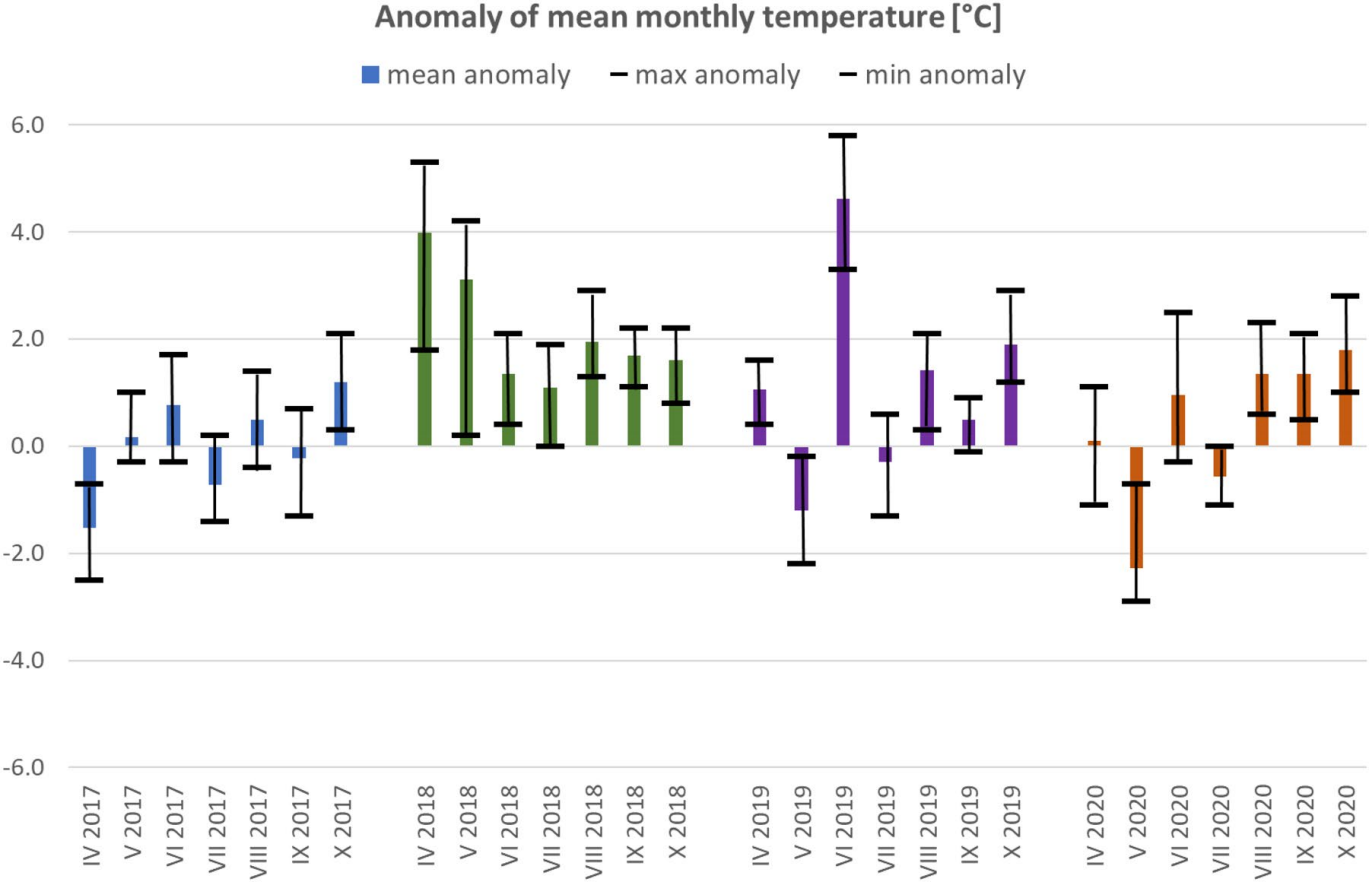
Ranges of anomaly of mean monthly temperature during the period 2017–2020 in reference to the climatological norm (1991–2020).

The analysed periods covering warm season (April–October) between 2017 and 2020 were characterised with various meteorological conditions with dominance of months with a positive thermal anomaly (referring to climate norm 1991–2020) (Fig. 8). The year 2017 represented the lowest thermal variability within the analysed period. In 2018, all the months analysed identified positive anomalies, with the highest value in April (+ 4.0 °C). The highest anomaly was observed in June 2019, reaching on average + 4.6 °C, and locally close to + 6.0 °C in the western part of Poland. The lowest temperatures were recorded in May 2020. This month of the year showed the greatest variability of thermal conditions from + 3.1 °C (May 2018) to − 2.3 °C (May 2020).

Veterinary medicine practitioners (i.e., animal owners and veterinarians) seem to lack knowledge on whether and which climate parameters should be used to predict disease occurrence and on the laboratory tests required to optimise animal health and productivity. Mathematical models are used to assess the non-linear relationships between pathogen transmission and observed temperature changes. Mechanistic models are used to predict the transmission of infectious viruses. Nonlinear mechanistic models can be used to predict non-vectored parasite transmission, as confirmed in wildlife^7^. Despite advances, none of these mathematical or epidemiological models is considered simple or gives direct hallmarks of practical needs for the control of animal health. The selection of simple and easily acquired data for such purposes opens up completely new possibilities for the training of breeders regarding which parameters merit their attention. Therefore, multifaceted cooperation is needed between veterinarians, meteorological research institutes, and agricultural advisory centres that serve breeders. The joint development of critical control points for meteorological data may contribute to an improved understanding of animal productivity and the reduction of animal laboratory testing costs.

Gastrointestinal (GI) nematode infection threatens the health, productivity, and welfare of dairy cattle and is thus a serious issue in cattle management. Controlling such parasite invasion requires not only proper grazing and farm management practises, but also comprehensive knowledge of its epidemiology and an understanding of the role of agroclimatic conditions such as temperature and rainfall^36^. The development of the free-living stages of GI parasites in pastures is highly dependent on temperature (warmer temperatures increase the development rate) and moisture^37–39^. Temperature also affects grass growth and therefore exposure to larvae, regardless of their developmental and survival rates^40^.

In this study, relationships between selected meteorological indicators and ODRs for ostertagiosis that may influence the milk production of dairy cattle in Poland located in central Europe were explored for the first time. Some kind of study that were identified on the effect of temperature and the development of the free-living stages of parasites, based on only the experimental study^41^. Bulk tank milk testing and ODR determination provide valuable indicators of herds’ antiparasitic response and potential production losses^42^. A high ODR indicates that the animals have been exposed to a high larval challenge in pasture, including current and earlier pasture seasons^43^. For ostertagiosis, ODRs > 0.5 may correlate with decreases in milk production of 0.5 to more than 2.5 kg/cow/day. Although serology is an excellent predictive tool, cost reduction (in light of rapid global inflation and low milk purchase prices) remains a priority for breeders. Thus, a method is needed to allow for more precise selection of herds for testing.

From the point of view of dairy production, a simple, routine laboratory examination of fecal samples is unprofitable and does not apply in field conditions. Comparing our own results with those reported in other studies, the same tendency of relationship of temperature and humidity dependence appeared. In the study by Pandey^41^, an optimal temperature of 25 °C was found for the development of infective larvae. Our results indicate that the selection of herds for farmers' testing can be guided by regular checking of meteorological data, especially since various temperature and humidity indicators require observation. This is particularly important for the months of the warm period of the year (IV–X) in Poland, when cows have access to pasture or feed with forages. There is a higher risk of contact with invasive larvae in the environment, as well as considering feeding with green fodder. In general, ranges of 24–28 °C, as well as − 0.5 to 7.5 °C for the monthly maximum and minimum temperature, respectively, and relative humidity (68–79%) and vapour pressure (10–15 hPa) correspond to a high antiparasitic response of the herd, expressed as the optical density ratio (ODR). From the point of view of inhibiting larvae development, beneficial effects were apparent if the air temperature exceeds 35 °C ^41^. In our study, we also found a strong, negative correspondence between very high ODR values and the maximum monthly air temperature exceeding 33 °C. We observed some tendencies taking into account air humidity and obtained ODR results. Most of the high ODRs values in our research seem to occur in the range of 68–79% or higher. In the experimental analysis of humidity and its influence on parasites by Pandey et al.^44^, 75% and 95% RH seem to reduce the survival rate of larvae.

In conclusion, based on the results obtained, some practical recommendations can be formulated. In the case that the observed weather pattern (covering the prepatent period of the parasite development-about 3 weeks) fits with the estimated weather ranges, coproscopical examination or ODR testing should be implemented. An increase in the intensity of invasion will determine the need for deworming to prevent a serious decrease in milk production.

We estimated ranges of climatological parameters suggesting the need for laboratory testing of herds and serving as predictors for further herd monitoring. This was confirmed with the 95% confidence level. Simple visualisation of trends in climate parameters based on publicly available weather information can facilitate breeder decision making about the need to test herds and contribute to improving dairy cattle management. An additional benefit would be to equip farms with a dedicated local meteorological station that could allow the individual farmers to monitor current meteorological conditions and apply necessary testing/ treatment of the herds.

Further development of measures for tracking climate and environmental changes contributing to parasite surviving and resistance requires gathering of real-time weather data of spatio-temporal coverage tailored to grazing areas and practises, developing new control measures, and early warning procedures.

## Materials and methods

### Design, sample collection, and assumption to include enzyme-linked immunosorbent assay (ELISA) results

According to Polish legal regulations, no formal approval of the bioethical commission was required on participation of cattle herd owners in the study, since the questionnaires applied to animal and not human health. Cattle herd owners gave their informed verbal consent to participate in the study. All methods were carried out according to relevant guidelines and regulations. Examination of BTM samples collected from the tanks does not require approval of the local ethics committee.

In total, 1111 dairy cattle farms with a risk of decreased milk production due to the presence of ostertagiosis and no regular or lack of deworming procedures were included in this study. Farmers took part in the research programme voluntarily and declared their participation in the project to veterinarians who cooperated with farms. They were also requested to fulfil questionnaire on herd (herds size, number of lactating animals, feeding, access to pasture and grazing policy, average milk yield, control of parasites, etc.).

The size of the herd ranged from 20 to 1300 adult Holstein–Friesian (HF) dairy cows. The maintenance system of the animals, as well as the feeding system, was related to the herd size and the milk production. For small herds (up to 100 lactating cows), animals had free access to pasture and water during the grazing period (“grazing”). For medium herds (> 100 < 500 lactating cow), depending on the organisational potential of the farm, a part of the animals may have access to pasture ('mix grazing'). In larger objects (> 500 lactating animals) cows have been maintained in the indoor keeping system (loose housing system or under the keeping system in a shallow cow-house; “no grazing”). Animal feed based on total mixed ration (TMR), including pasture grass forage, grains, protein feeds, minerals, vitamins, and feed additives. Machine milking has been applied in all farms, whereby for parts of large farms, the milking units were wirelessly connectable to the monitoring dairy management system, automatically sending and receiving valuable data during the milking process. Detailed milk performance and SCC were available only for owners covered by the Milk Quality Control Programme for Dairy Cattle implemented by the Polish Federation of Cattle Breeders and Milk Producers. The deworming programme was only applied to calves in large herds, while no treatment was carried out on animals in small and medium herds.

Bulk tank milk (BTM) samples were collected by local veterinarians from the bulk tanks located on farms in 16 voivodeships of Poland. Each herd was visited once between 2016 and 2020 and the off-grass period (XI-III) was excluded from the analyses. BTM samples were stored in 100-ml containers without preservatives and transported at 4 °C to the EPIVET Diagnostic Laboratory (Faculty of Veterinary Medicine, Wroclaw University of Environmental and Life Sciences). Immediately upon receipt, the samples were prepared for the measurement of *Ostertagia ostertagi* antibody level using a semiquantitative indirect enzyme-linked immunosorbent assay commercial (ELISA; SVANOVIR®, Svanova, Sweden), as previously described^45^. The milk was centrifuged for 15 min. at 1200×*g* and then the fat fraction was carefully moved. Skimmed milk samples were used directly for analysis or stored at − 20 °C until testing. The inclusion criterion for the analysis of weather parameters was *Ostertagia ostertagi* herd infection, confirmed by indirect bulk tank milk testing. According to the manufacturer's instructions, ODR levels 0.5 were taken to correspond as the 'cut-off' value to potential losses in milk production. The pasture period in Poland usually lasts from April to October. In the time when animals are kept closed, meteorological conditions have no direct impact on the development of the invasive stages of parasites in the environment. Therefore, finally, the 671 herds were investigated after excluding the off-grass period (XI-III) (see supplementary materials).

### Calculation of climate parameters

**Figure 9:**
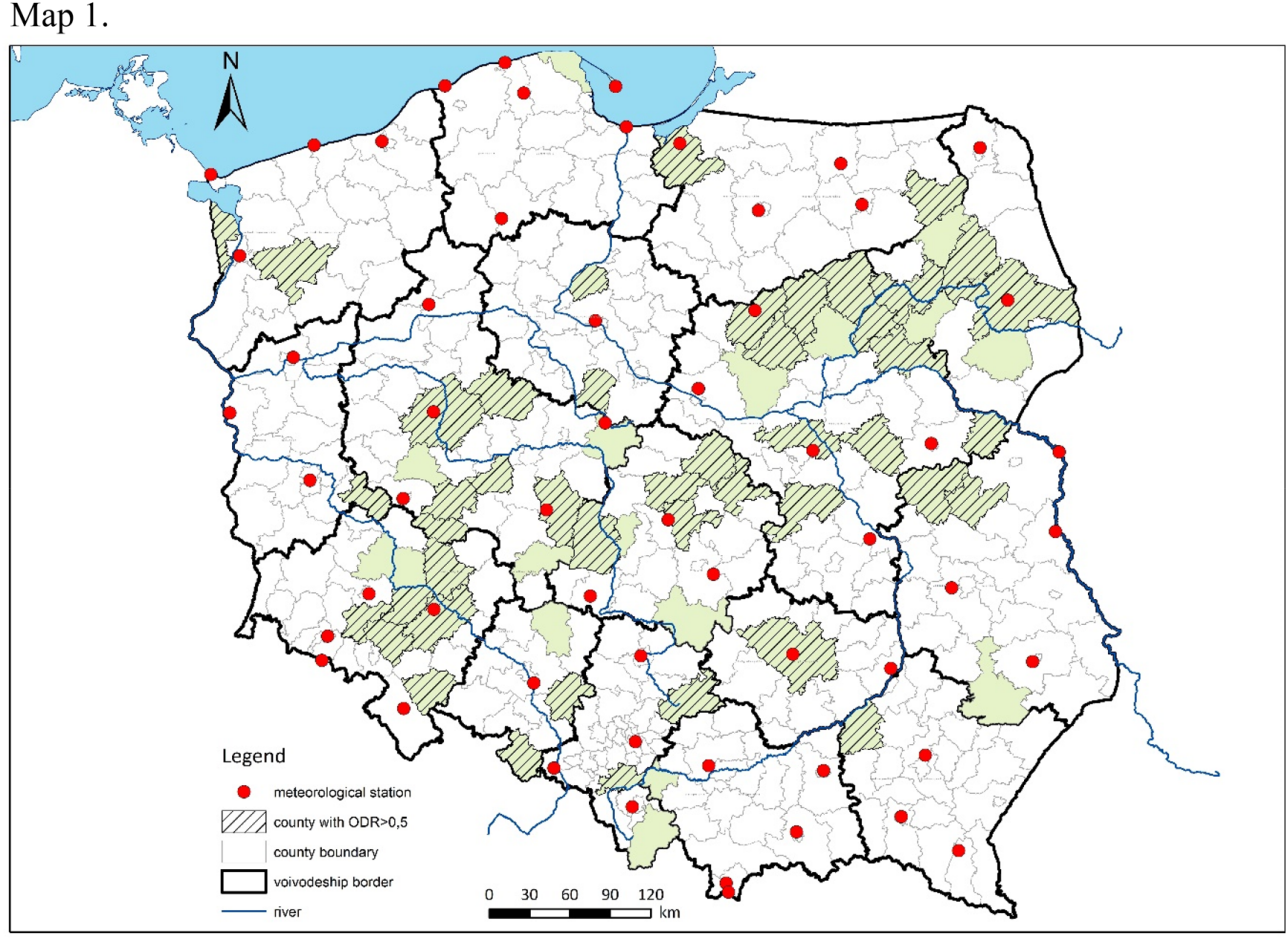
Locations in Poland of sample collection (green areas) and weather stations from which data were obtained (red dots). Hatching indicates districts with ODR values ≥ 0.5 during the period 2017–2020.

Meteorological data were obtained from 57 monitoring stations operated by the Institute of Meteorology and Water Management of the National Research Institute (Fig. 9). Based on daily data, the following climatic indicators were calculated: mean monthly air temperature(Ta) (°C), monthly absolute maximum and minimum air temperatures (Tx_ab, Tn_ab) (°C), mean monthly temperatures of daily minima (Tn) and maxima (Tx) (°C) and mean monthly relative humidity (RH) (%), mean monthly vapour pressure (VP) (hPa).

**Figure 10:**
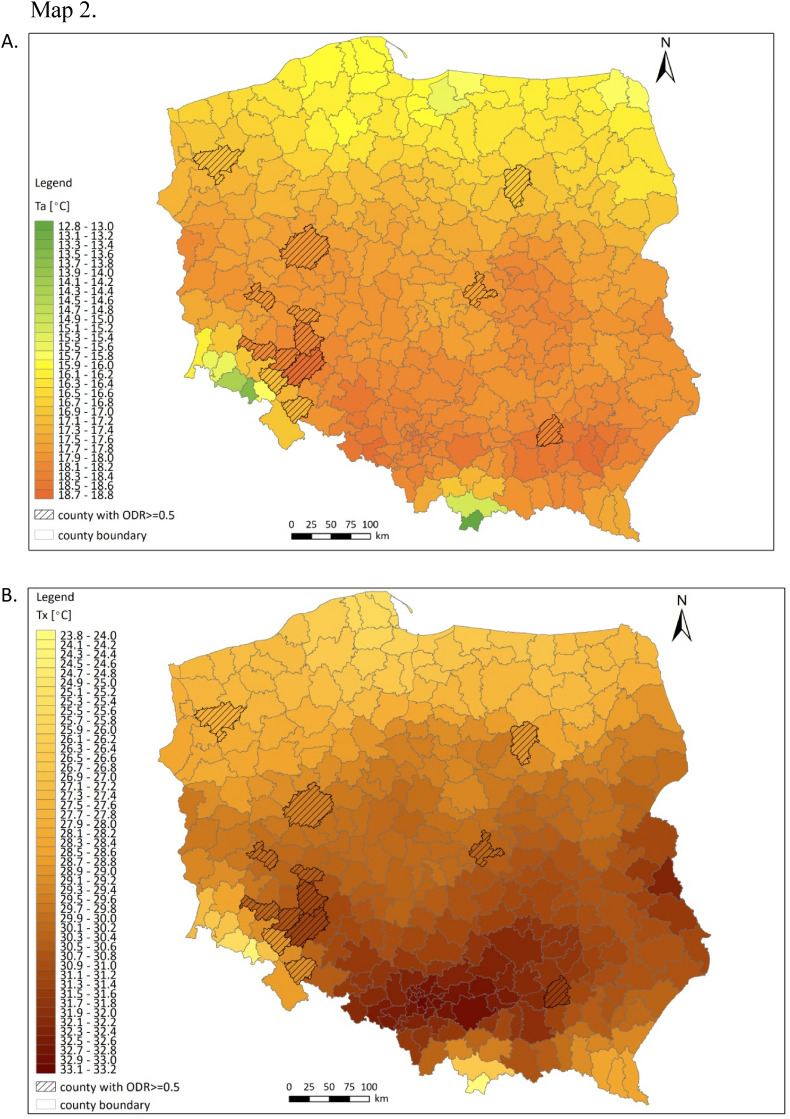
Regional mean monthly air temperatures (Ta, **A**) and mean monthly temperatures of daily maxima (Tx, **B**), and locations of districts in Poland with ODR values ≥ 0.5 (hatching); example for June 2017.

These values were spatially interpolated using inverse distance weighting (IDW) for each month of the warm season of the year (April–October) for the period 2017–2020. The resulting gridded database was used to estimate the values of the selected indicators by district (Fig. 10).

The distribution of administrative districts in which ODR values were examined is presented on Fig. 9. Each combination of ODRs was addressed to the values of the climatic indicators as binary events (present or absent).

### Database creation

To evaluate the associations between the BTM ELISA results and the selected climate parameters, a database was created. For each BTM sample, an ODR value was assigned together with the values of the climate parameter characterizing the month of sample collection at the farm site (Table 1).

**Table 1:** An example of the input data inventory.

Year	Month	Province	District	Ostertagia ODR	Tx_ab (°C)	Tx (°C)	Tn_ab (°C)	Tn (°C)	Ta (°C)	RH (%)	VP (hPa)
2017	5	Dolnośląskie	Lubiński	0.434	29.9	20.0	− 0.4	8.2	14.1	72.9	11.7
2017	5	Dolnośląskie	Lubiński	0.072	29.9	20.0	− 0.4	8.2	14.1	72.9	11.7
2017	5	Dolnośląskie	Trzebnicki	0.097	31.2	20.7	1.0	9.3	15.0	69.7	11.7
2017	5	Dolnośląskie	Trzebnicki	0.268	31.2	20.7	1.0	9.3	15.0	69.7	11.7
2017	5	Dolnośląskie	Trzebnicki	0.123	31.2	20.7	1.0	9.3	15.0	69.7	11.7
2017	5	Dolnośląskie	Trzebnicki	0.336	31.2	20.7	1.0	9.3	15.0	69.7	11.7
2017	5	Dolnośląskie	Trzebnicki	0.110	31.2	20.7	1.0	9.3	15.0	69.7	11.7
2017	5	Dolnośląskie	Trzebnicki	0.617	31.2	20.7	1.0	9.3	15.0	69.7	11.7
2017	5	Dolnośląskie	Trzebnicki	0.256	31.2	20.7	1.0	9.3	15.0	69.7	11.7
2017	6	Dolnośląskie	Świdnicki	0.255	32.0	25.6	8.0	13.2	19.3	64.4	14.0

### Data analysis

To identify parameters that can serve as predictors for selective laboratory-based herd monitoring, the established database containing climatological and respective ODR values was examined based on the construction of contingency tables. Two levels of high ODR values (≥ 0.5 and ≥ 0.8) was assessed in combination with the value of the climatological parameter while changing the threshold level iteratively by 0.1 °C for temperature indicators, 1% for relative humidity and 0.1 hPa for the pressure of vapour.

The Yule *Q* coefficient was used to assess associations between the presence of high ODR values and the presence of the respective climatological parameters that exceeded the investigated threshold levels (Table 2).

**Table 2:** Construction of contingency tables that meet the assumed conditions on ODR and climatological parameters.

Observed frequencies		Climatological parameter (CP)	
		CP ≥ threshold	CP < threshold
ODR	ODR ≥ 0.5 (0.8)	O_11_	O_12_
	ODR < 0.5 (0.8)	O_21_	O_22_

This coefficient is used to assess the relationships between qualitative characteristics, with the data entered into 2 × 2 contingency tables^46^. The observed frequencies *Oij* (*i* = 1*,* 2*, r*; *j* = 1*,* 2) represented the occurrence of both features. *The Q* coefficients (range, − 1 to 1) were determined using the formula.

$$Q = \frac{{O_{11} O_{22} - O_{12} O_{21} }}{{O_{11} O_{22} + O_{12} O_{21} }}$$.

Values closer to 0 indicate weaker dependence of the features, and those closer to − 1 or 1 indicate stronger dependence. The statistical significance of the coefficients was verified using the *Z* test, with *Z* defined as:

$$Z = \frac{Q}{{\sqrt {\frac{1}{4 }(1 - Q^{2} )^{2} \left( {\frac{1}{{O_{11} }} + \frac{1}{{O_{12} }} + \frac{1}{{O_{21} }} + \frac{1}{{O_{22} }}} \right)} }}.$$

*The P* values designated based on the *Z* statistics were considered significant at ≤ 0.05. All the calculations and calculations were done with the use of R programming language, including Verification library.

## Supplementary Information

Supplementary Information.

## Data Availability

Raw data used in the production of the tables and figures are available in one of the authors (wiwiana.szalinska@imgw.pl) upon request.
